# The transcription factor ZNF683 marks an exhaustion-like GZMB^+^CD8^+^ T cell in sepsis

**DOI:** 10.3389/fimmu.2026.1756339

**Published:** 2026-03-06

**Authors:** Mingtong Hou, Zhao Mi, Shiyu Ouyang, Wenbo Wang, Guiquan Zhao, Shengbao Wang

**Affiliations:** 1 The Second Clinical Medical School, Lanzhou University, Lanzhou, Gansu, China; 2 Emergency Center, Lanzhou University Second Hospital, Lanzhou University, Lanzhou, Gansu, China

**Keywords:** GZMB, immunosuppression, lag3, sepsis, T cell exhaustion, ZNF683

## Abstract

**Background:**

Sepsis is a life-threatening syndrome caused by a dysregulated host response to infection and remains a major global cause of mortality. Persistent immunosuppression contributes to secondary infections and adverse outcomes, yet the mechanisms underlying late-phase T-cell dysfunction remain incompletely understood.

**Methods:**

We integrated publicly available human peripheral blood mononuclear cell single-cell RNA sequencing with a clinically relevant cecal ligation and puncture (CLP) mouse model to characterize CD8^+^ T-cell states during sepsis. Key computational findings were supported by flow cytometry and RNA fluorescence *in situ* hybridization (RNA FISH). The immunophenotypic effects of LAG3 blockade were evaluated in septic mice.

**Results:**

Single-cell analysis identified a GZMB^+^CD8^+^ T-cell population with an exhaustion-like transcriptional program in sepsis, characterized by increased expression of inhibitory receptors including LAG3 and elevated ZNF683. ZNF683 expression tracked with exhaustion-associated features within the CD8^+^GZMB^+^ compartment. In CLP mice, anti-LAG3 treatment partially improved frequency of GZMB^+^CD8^+^ T cells by flow cytometry. RNA FISH further showed reduced ZNF683 signals in the lungs and liver of septic mice following LAG3 blockade.

**Conclusion:**

ZNF683 is associated with an exhaustion-like GZMB^+^CD8^+^ T cell state in sepsis and may contribute to persistent T-cell dysfunction. Further mechanistic studies directly perturbing ZNF683 are needed to determine its causal role and therapeutic potential.

## 1 Introduction

Sepsis, defined as life-threatening organ dysfunction caused by a dysregulated host response to infection (1), poses a major global health burden, affecting approximately 50 million individuals and causing about 5.3 million deaths each year (2). Survivors are at increased risk of readmission, with rates exceeding those of non-sepsis hospitalizations; notably, a substantial proportion of readmissions are attributed to new infections (3, 4). This clinical trajectory, together with the rising incidence of sepsis, highlights the need to better understand the persistent immunosuppressive state that increases susceptibility to infections after the acute episode.

The pathogenesis of sepsis involves a complex interplay of inflammatory dysregulation, immune dysfunction, and other pathophysiological processes that culminate in organ failure (5–7). An initial hyperinflammatory “cytokine storm” is frequently followed by a prolonged phase of immune suppression, characterized by impaired pathogen clearance and increased vulnerability to secondary opportunistic infections (8). Sepsis-induced immunosuppression involves both innate and adaptive immune compartments and can directly or indirectly impair the function of multiple immune cell types (9–12).

CD8^+^ T cells are essential for the control and eradication of intracellular pathogens. Upon antigen encounter, naïve CD8^+^ T cells differentiate into effector cells with cytolytic activity and cytokine-producing capacity (4, 13, 14). In sepsis, however, lymphocyte apoptosis leads to marked depletion of circulating T cells, and surviving CD8^+^ T cells can exhibit early and sustained functional impairment, which may contribute to increased susceptibility to both recurrent and new infections (15, 16). A clearer understanding of how sepsis reshapes CD8^+^ T-cell function is therefore important for elucidating the mechanisms of post-sepsis immune vulnerability.

T cell exhaustion is a state of T cell dysfunction that develops in settings of persistent antigen stimulation, such as chronic infections and cancer. It is characterized by reduced effector function and proliferative capacity, together with increased expression of inhibitory immune checkpoint receptors (17). Studies in patients with sepsis have reported quantitative and functional alterations in circulating effector CD8^+^ T cells, including impaired cytokine production and diminished responsiveness to secondary infection challenges (7, 18–22). Although clinical evidence supporting therapies targeting T cell exhaustion–related immune checkpoints such as PD-1, TIM-3, LAG-3, and CTLA-4 in sepsis remains limited, numerous preclinical models have demonstrated that blockade of these inhibitory checkpoints can restore both innate and adaptive immune cell functions, enhance host resistance to infection, and improve survival in some preclinical sepsis models (23). Collectively, these observations implicate exhaustion-like T-cell dysfunction as a contributor to sepsis-associated immunosuppression and adverse outcomes (24, 25).

ZNF683, also known as Hobit (homolog of Blimp-1 in T cells), is a transcription factor involved in the differentiation and tissue residency of cytotoxic lymphocytes, including NK cells and CD8^+^ T cells (26). In humans, ZNF683 is preferentially expressed in effector CD8^+^ T cells rather than naïve or most memory subsets (27). In a simian immunodeficiency virus model, ZNF683 has been linked to enhanced CD8^+^ T-cell proliferation and IFN-γ secretion, with concomitant suppression of viral replication (28). In several tumour microenvironments, ZNF683 marks CD8^+^ T-cell populations associated with anti-tumour immunity (29–31). Despite these findings, the role of ZNF683 in sepsis and its relationship to exhaustion-like CD8^+^ T-cell dysfunction remain poorly defined, and mechanistic evidence in clinically relevant sepsis models is limited.

As the risk of late morbidity and non-sepsis-related mortality increases among survivors years after the initial episode (32), the long-term immunological consequences of sepsis have become a major focus of recent research. However, how late-stage sepsis shapes CD8^+^ T cell states—particularly exhaustion-like programs—and the potential contribution of transcriptional regulators such as ZNF683 remain incompletely understood. Here, we integrated single-cell transcriptomic analysis of human peripheral blood immune cells with a polymicrobial cecal ligation and puncture (CLP) mouse model, complemented by flow cytometry and RNA fluorescence *in situ* hybridization, to characterize exhaustion-like CD8^+^ T cell states in advanced sepsis and to explore the association between ZNF683 expression and T-cell dysfunction. These findings may provide a rationale for future studies targeting inhibitory signalling pathways and transcriptional regulators to mitigate sepsis-associated immunosuppression.

## 2 Materials and methods

### 2.1 Source of raw data

Single-cell RNA sequencing (scRNA-seq) data were obtained from the Gene Expression Omnibus (GEO; https://www.ncbi.nlm.nih.gov/geo/) and analyzed using the Seurat package in R (version 4.2.3). The dataset included late-stage sepsis patients diagnosed 14-21 days after sepsis (GSE175453), along with healthy individuals as controls.

### 2.2 Data processing of 10×scRNA-seq

Single-cell RNA-sequencing (scRNA-seq) data from sepsis and healthy control samples were processed using Seurat. Raw 10× Genomics count matrices were imported and converted into Seurat objects, followed by stringent quality control to remove low-quality cells and potential doublets. Cells were retained for downstream analysis if they met all of the following criteria: (i) 200–5,000 detected genes (nFeature_RNA), (ii) <20,000 total UMIs (nCount_RNA), and (iii) <15% mitochondrial transcripts (percent.mt). After filtering, 35,644 high-quality cells were retained for subsequent analyses.

For batch-effect correction across samples, we integrated datasets using Seurat’s canonical correlation analysis (CCA)–based anchor workflow. Briefly, each sample was normalized with NormalizeData and HVGs were identified using FindVariableFeatures (method = “vst”, nfeatures = 2000). Integration anchors were then identified using FindIntegrationAnchors (dims = 1:30), and an integrated expression matrix was generated with IntegrateData. The integrated data were subsequently scaled using ScaleData and subjected to principal component analysis (RunPCA). Two-dimensional embeddings were generated using RunUMAP (dims = 1:50), and graph-based clustering was performed using FindNeighbors (dims = 1:50) followed by FindClusters (resolution = 0.8).

To identify marker genes in various clusters, FindAllMarkers was applied with thresholds of |log2FC| ≥ 0.3 and min.pct ≥ 0.25 (Wilcoxon rank-sum test; p_val_adj < 0.05). Cell type identification was performed based on the expression of canonical marker genes derived from published literature and the CellMarker 2.0 database (33). The corresponding markers used for each immune cell population are summarized in **Table 1**. Specifically, the expression of representative genes was used to define major immune cell types, including T cells (CD3D, CD3E, CD3G, IL7R), plasma cells (CD27, CD28, MZB1), plasmacytoid dendritic cells (pDCs; DERL3, IL3RA, LILRA4, CLEC4C), natural killer (NK) cells (XCL1, NCAM1, GNLY, KLRF1), neutrophils (S100P, LCN2, LTF), monocytes (FCGR3A, CD14, VCAN, FCN1), platelets (PPBP, TUBB1, GP9), mast cells (CNRIP1, CPA3, CD34), conventional dendritic cells (cDCs; CD1C, FCER1A, CLEC10A), and B cells (CD22, CD79A, MS4A1) (**Table 1**).

**Table 1:** summarizes the canonical marker genes used for the annotation of major immune cell types in this study. These markers were selected based on CellMaker 2.0.

Cell type	Representative markers
T cells	CD3D, CD3E, CD3G, IL7R
Plasma cells	CD27, CD28, MZB1
Plasmacytoid dendritic cells (pDCs)	DERL3, IL3RA, LILRA4, CLEC4C
Natural killer (NK) cells	XCL1, NCAM1, GNLY, KLRF1
Neutrophils	S100P, LCN2, LTF
Monocytes	FCGR3A, CD14, VCAN, FCN1
Platelets	PPBP, TUBB1, GP9
Mast cells	CNRIP1, CPA3, CD34
Conventional dendritic cells (cDCs)	CD1C, FCER1A, CLEC10A
B cells	CD22, CD79A, MS4A1

We removed cell clusters that expressed two or more major lineage markers on the UMAP during clustering, which were considered potential mixed-lineage/doublet-like clusters.

### 2.3 T-cell reanalysis

We analysed the T-cell compartment separately by subsetting T cells based on canonical markers. The subset was re-normalised using NormalizeData (method = “LogNormalize”, scale factor = 10,000), HVGs were identified using FindVariableFeatures, and PCA was performed using RunPCA. UMAP visualisation and graph-based clustering were conducted using the first 50 PCs (RunUMAP/FindNeighbors, dims = 1:50; FindClusters, resolution = 0.8).

### 2.4 Phenotypic characterization of CD8^+^ T cells

To characterize the phenotypic profile of CD8^+^ T cells, we selected a panel of reference markers, including exhaustion markers (PDCD1, TIGIT, CTLA4, TNFRSF9, HAVCR2, LAG3) and cytokines/effector molecules (CST7, GZMK, GZMA, NKG7, IFNG, PRF1, GZMB, GNLY), which are widely recognized indicators of T cell activation, exhaustion, and effector function.

### 2.5 KEGG and GO enrichment analysis

Differentially expressed genes between CD8_Tex_GZMB^+^LAG3^+^ cells and CD8_Tem_GZMB^+^LAG3^−^ cells were identified using FindMarkers (Wilcoxon rank-sum test; p_val_adj < 0.05). Subsequently, Gene Ontology (GO) and Kyoto Encyclopedia of Genes and Genomes (KEGG) enrichment analyses were performed using the clusterProfiler package (34).

### 2.6 Cell–cell interaction network analysis

Cell–cell communication analysis was performed using CellChat (v1.6.1) to infer ligand–receptor interactions (35). Analyses were conducted on the RNA assay (log-normalized expression) together with curated cell-type annotations. We restricted the analysis to T-cell subsets (CD4/CD8 subclusters) to interrogate within–T-cell communication. The CellChatDB.human database was used. Communication probabilities were estimated using computeCommunProb (default settings) and filtered with filterCommunication(min.cells = 10) to remove interactions supported by low-abundance cell groups. Pathway-level communication probabilities were computed using computeCommunProbPathway and summarized with aggregateNet. Network visualizations were generated from aggregated pathway probabilities; edge.weight.max was adjusted only for visualization comparability and did not affect the underlying inference.

### 2.7 Pseudotime trajectory analysis

To investigate the exhaustion trajectory of CD8^+^GZMB^+^ T cells during sepsis, pseudotime analysis was performed on three clusters (CD8_Naive, CD8_Tem_GZMB^+^LAG3^−^, and CD8_Tex_GZMB^+^LAG3^+^) using Monocle2 (36). Ordering genes were defined as the union of differentially expressed genes identified among these three clusters using Seurat FindMarkers (Wilcoxon rank-sum test; p_val_adj < 0.05). Trajectories were then constructed using reduceDimension and orderCells with default settings, and gene-expression dynamics were assessed along the inferred pseudotime.

### 2.8 Animal experiments and CLP-induced sepsis

Eighteen male C57BL/6 mice (6–8 weeks old, weighing 22–28 g) were obtained from GemPharmatech Co., Ltd. and housed in standard cages under a 12-hour light/dark cycle. Ambient temperature was maintained at 24 ± 1 °C, and relative humidity was controlled at 40–60%. Mice had ad libitum access to standard chow and water. All experimental procedures were approved by the Laboratory Animal Ethics Committee of the Second Hospital of Lanzhou University (Approval No. D2025881).

Mice were randomly allocated into three groups (n = 6 per group): Sham, CLP, and Treatment. The sepsis model was established using a previously described method (37). Sham mice underwent laparotomy without cecal ligation or puncture. CLP mice underwent cecal ligation and puncture surgery to induce polymicrobial sepsis. Treatment mice received 40 µg anti-mouse LAG-3 antibody (HY-P99141, MedChemExpress) via tail vein injection once daily for three consecutive days prior to CLP and for two consecutive days after CLP.

The sepsis mouse model was established using CLP. After anesthetizing mice with isoflurane, a midline abdominal incision was made to expose the cecum. The upper one-third of the cecum was ligated using a 4-0 silk suture, and the distal cecum was punctured transversely with a 21-gauge needle. A small amount of fecal content was gently extruded to ensure patency, the cecum was returned to the abdominal cavity, and the abdomen was closed in layers. A sham-operated group (Sham) was also included, in which mice underwent identical anesthesia and laparotomy procedures to expose the cecum, but without ligation or puncture, followed by layered closure of the abdomen.

All analyses were performed 48 h after CLP. Mortality occurred prior to the planned endpoint: no deaths occurred in the Sham group; three mice died in the CLP group (50% mortality), whereas one death occurred in the Treatment group. For Sham and Treatment groups, three mice were randomly selected from survivors at 48 h for endpoint assays to match group sizes; for the CLP group, all surviving mice were included. Because downstream immunophenotyping was performed on survivors at 48 h, the results may be influenced by survivorship bias. Blood samples were collected via cardiac puncture; spleens were harvested for flow cytometric analysis; and liver and lung tissues were collected for histological analysis and fixed in 4% paraformaldehyde (PFA).

### 2.9 Flow cytometry

To assess surface molecule expression, cells were stained in FACS buffer for 40 min with FITC anti-mouse CD45 (Biolegend, 103107), APC anti-mouse CD3 (Biolegend, 100235), PerCP/Cyanine5.5 anti-mouse CD8a (Biolegend, 100733), and PE anti-mouse CD223 (LAG-3) monoclonal antibody (Invitrogen, 12-2239-42). For intracellular staining, cells were permeabilized and incubated with Alexa Fluor^®^700 anti-mouse Granzyme B recombinant antibody (Biolegend, 372222). Flow cytometric analysis was performed using a BD FACSCanto cytometer, and raw data were collected. Data were analyzed using FlowJo software.

### 2.10 Fluorescence *in situ* hybridization

Liver and lung tissues were excised, washed with PBS, and fixed in 4% paraformaldehyde (PFA). Tissues were sectioned at 4 μm, mounted on glass slides, deparaffinized with xylene, and rehydrated through a graded ethanol series (30–100%). Hybridization solution containing a 1:20 dilution of ZNF683 FISH probe was added dropwise, and slides were incubated overnight at 42 °C. For RNA FISH, fixed and permeabilized slides were treated with HRP-conjugated Avidin (1:100 dilution in PBS) for 15 min, followed by TSA amplification for 15 min to enhance fluorescence signal sensitivity. Nuclei were counterstained with DAPI for 5 min, and slides were examined using a confocal microscope.

### 2.11 Statistical analysis

All analyses were performed in R (v4.2.3). For single-cell RNA-seq analyses, statistical testing was conducted using Seurat and related R packages, and multiple-testing correction was applied where appropriate; adjusted p values < 0.05 were considered statistically significant for differential expression analyses. For mouse experiments, due to the small sample size (n = 3 per group for endpoint readouts) and the lack of assurance of normality, non-parametric tests were used. Overall differences among groups were assessed using the Kruskal–Wallis test, followed by Dunn’s *post hoc* test with BH correction for multiple comparisons.

## 3 Results

### 3.1 Ten distinct cell populations were identified in peripheral blood immune cells from patients with sepsis using scRNA-seq data analysis.

**Figure 1:**
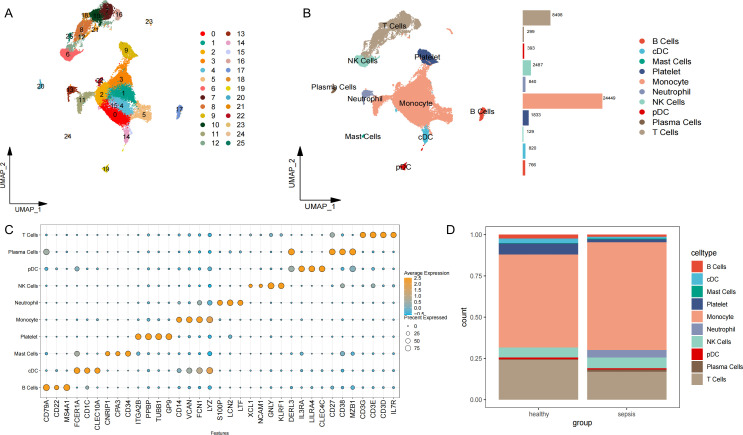
Single-cell transcriptome profiling of peripheral blood mononuclear cells (PBMCs) from patients with late-stage sepsis and healthy volunteers. **(A, B)** Uniform Manifold Approximation and Projection (UMAP) visualizations of PBMCs from healthy volunteers (Healthy; n = 5) and patients with sepsis (Sepsis; n = 4), clustered based on the expression levels of marker genes. **(C)** Expression of characteristic marker genes across all cell types. **(D)** Cellular composition and proportional distribution across groups.

The scRNA-seq dataset GSE175453 was retrieved from the Gene Expression Omnibus (GEO) database to construct a systematic transcriptomic atlas of sepsis at the single-cell level. This dataset comprised peripheral blood immune cells generated from a mixture of whole blood myeloid-enriched and Ficoll-enriched peripheral blood mononuclear cells (PBMCs) obtained from four patients with late-stage sepsis and five healthy volunteers. Following quality control and normalization (**Supplementary Figure 1**), cells were clustered using a graph-based approach and visualized by UMAP, yielding 26 transcriptionally distinct clusters (**Figure 1A**). Subsequent cell annotation was performed using known cell-specific marker genes (**Figure 1B**). A dot plot (**Figure 1C**) illustrates the expression patterns of key marker genes across the identified cell types. Based on these annotations, ten immune cell populations were identified in peripheral blood immune cells: monocytes, T cells, plasmacytoid dendritic cells (pDCs), platelets, mast cells, conventional dendritic cells (cDCs), natural killer (NK) cells, plasma cells, B cells and neutrophils.

The relative proportions of these cell populations varied between healthy controls and patients with sepsis, as shown in **Figure 1D**. Notably, sepsis samples showed a marked reduction in T-cell representation and a relative increase in neutrophils compared with healthy controls (**Figure 1D**), consistent with profound immune dysregulation in late-stage sepsis.

### 3.2 T cells were categorized into seven major subsets and a LAG3-high exhausted-like population emerged within CD8^+^GZMB^+^ cells.

**Figure 2:**
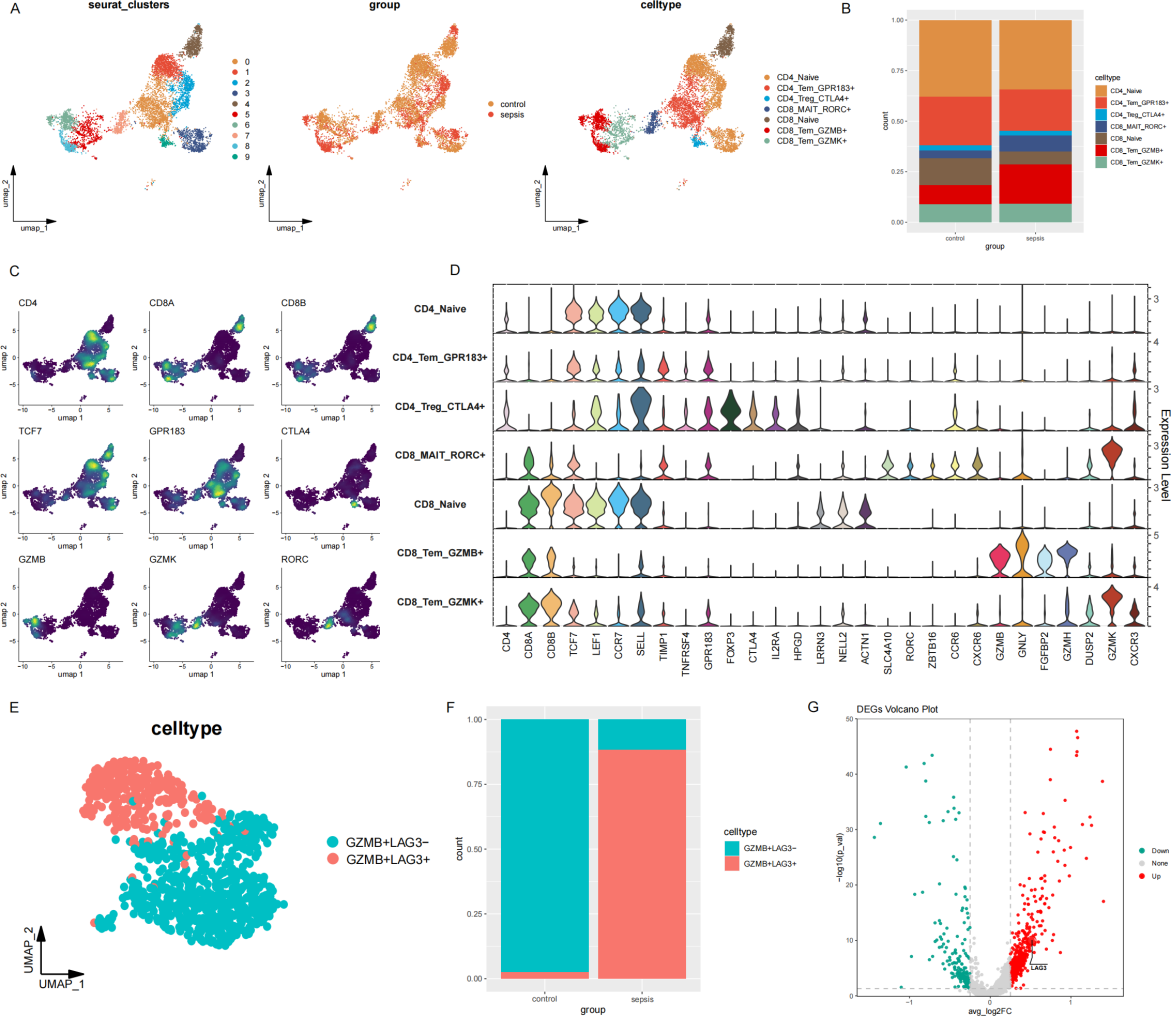
Single-cell analysis of T cell subpopulations. **(A)** Reference UMAP of T cells. **(B)** Proportions of T cell subpopulations across experimental groups. **(C)** Expression levels of indicated genes visualized per cell. **(D)** Violin plots stacked by T cell subset, showing expression of characteristic marker genes. **(E)** scRNA-seq profiles of GZMB^+^CD8^+^ T cells isolated from the total T cell population. Cells were stratified into LAG3^+^ and LAG3^-^ GZMB^+^CD8^+^ T cell populations based on LAG3 expression, a signature marker for exhausted CD8^+^ T cells. **(F)** Prevalence of LAG3^+^ and LAG3^-^ GZMB^+^CD8^+^ T cell populations in control subjects and sepsis patients. **(G)** Volcano plot displaying differentially expressed genes (DEGs) between LAG3^+^ and LAG3^-^ GZMB^+^CD8^+^ T cell populations. LAG3 is highlighted. DEGs were defined by a threshold of |log2FC| > 0.25 and an adjusted P-value < 0.05.

To investigate CD8+ T cell exhaustion in sepsis, we first characterized the T cell landscape (**Figures 2A, B**). Cells were clustered into seven subsets based on established marker genes (**Figures 2C, D**): CD4_Naive, CD4_Tem_GPR183^+^, CD4_Treg_CTLA4^+^, CD8_MAIT_RORC^+^, CD8_Naive, CD8_Tem_GZMB^+^, and CD8_Tem_GZMK^+^. Subpopulations exhibiting apparent quantitative differences between healthy controls and septic patients were prioritized for further analysis. Among these, the CD8_Tem_GZMB^+^ subset was preferentially represented in septic patients (**Figure 2B**) and displayed elevated expression of cytotoxicity-related genes, including GZMA, GNLY, FGFBP2, and GZMH (**Figure 2D**).

To explore heterogeneity within this cytotoxic CD8^+^ compartment, we re-analysed the CD8_Tem_GZMB^+^ population. After reclustering and dimensionality reduction, cells separated into two transcriptionally distinct subgroups according to LAG3 expression: CD8_Tem_GZMB^+^LAG3^-^ and CD8_Tex_GZMB^+^LAG3^+^ (**Figures 2E, F**). The LAG3-high subgroup displayed a distinct transcriptional profile relative to the LAG3^-^ subgroup (**Figure 2G**), consistent with an exhausted-like state.

### 3.3 CD8_Tex_GZMB^+^LAG3^+^ cells exhibit reduced cytotoxic programs and an exhausted-like functional profile in late-stage sepsis.

**Figure 3:**
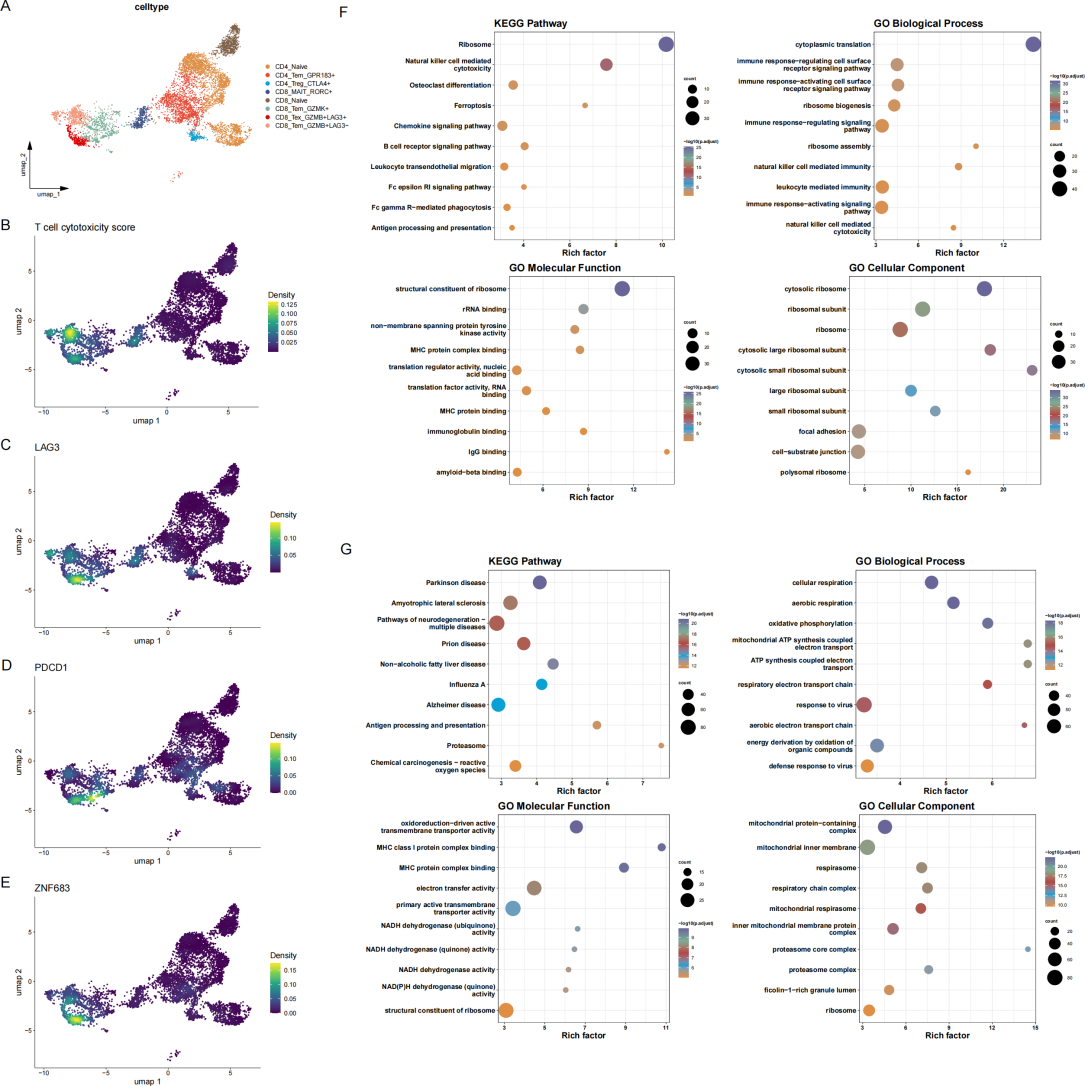
**(A)** Reference mapping of T cells showing the overall distribution of major subsets. **(B–E)** Cells were scored for the expression of cytotoxicity-associated genes **(B)** and exhausted CD8^+^ T cell signatures, including LAG3 **(C)**, PDCD1 **(D)** and ZNF683 **(E)**. All cells were projected onto a two-dimensional UMAP space to visualize transcriptional heterogeneity. **(F, G)** Bubble plots displaying the Gene Ontology (biological process [BP], cellular component [CC], and molecular function [MF]) and KEGG pathway enrichment analyses of differentially expressed genes (DEGs) between CD8_Tex_GZMB^+^LAG3^+^ cells and other CD8^+^ T cell subsets, identified using the “FindMarkers” function.

To further characterize the immune function of CD8_Tex_GZMB^+^LAG3^+^ cells in sepsis, GO and KEGG enrichment analyses were performed on differentially expressed genes between CD8_Tex_GZMB^+^LAG3^+^ cells and CD8_Tem_GZMB^+^LAG3^−^ cells. The upregulated genes in CD8_Tex_GZMB^+^LAG3^+^ cells were predominantly enriched in pathways associated with energy metabolism and antigen processing and presentation, whereas the downregulated genes were mainly related to cytotoxic effector functions. To quantitatively assess cytotoxic potential, the UCell scoring method was applied using established cytotoxicity-related gene signatures. CD8_Tem_GZMB^+^LAG3^−^ cells exhibited significantly higher cytotoxicity scores compared with CD8_Tex_GZMB^+^LAG3^+^ cells (**Figure 3B**), consistent with the reduced expression of multiple cytotoxic genes in the latter population. In addition, density visualizations provide an intuitive view of the single-cell co-expression patterns of GZMB with LAG3, PDCD1, and ZNF683 across the entire T-cell compartment (**Figures 2C**, **3C–E**). These plots highlight concordant expression patterns of LAG3, PDCD1, and ZNF683 at the single-cell level, consistent with an association among these markers within T cells.

**Figure 4:**
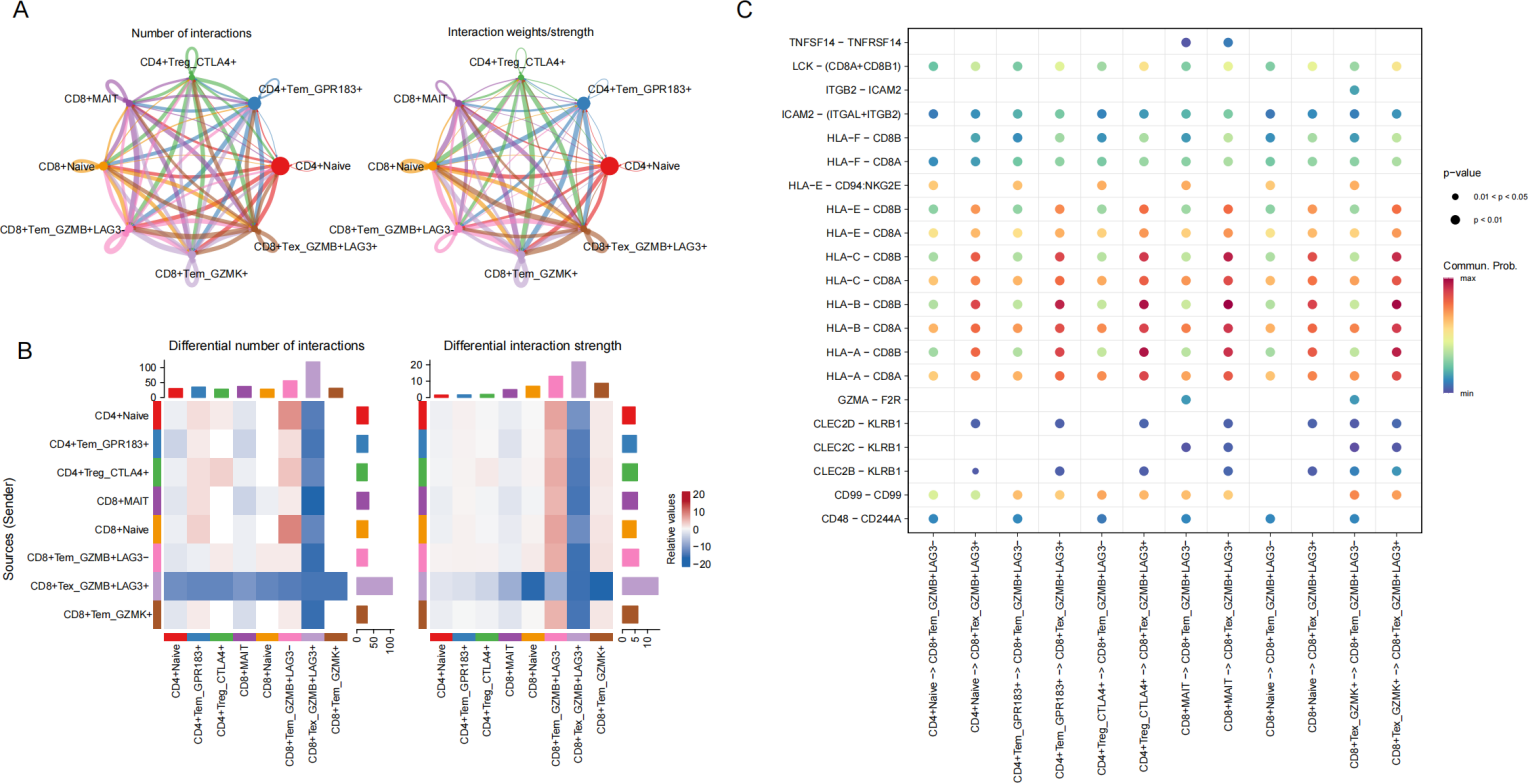
**(A)** Circular network diagram illustrating the number and strength of inferred intercellular communications among T-cell subsets. **(B)** Heatmap showing the relative abundance and intensity of potential ligand–receptor interactions between T-cell populations. **(C)** Bubble plot showing ligand–receptor pairs with higher inferred communication probabilities involving CD8_Tex_GZMB^+^LAG3^+^ cells compared with CD8_Tem_GZMB^+^LAG3^−^ cells within the T-cell compartment in late-stage sepsis samples.”.

To explore intercellular communication, pathway-level communication probabilities were inferred using CellChat by summarizing ligand–receptor interaction likelihoods across signaling pathways (**Figure 4A**). We restricted CellChat analyses to annotated T-cell subsets to examine within–T-cell communication patterns. Given the complexity of these networks, signaling patterns transmitted by each subset were visualized separately, and the edge.weight.max parameter was adjusted to enable comparison of interaction strengths across networks. The resulting communication maps revealed a reduction in both the number and intensity of interactions involving CD8_Tex_GZMB^+^LAG3^+^ cells relative to other T cell subsets (**Figure 4B**).

Furthermore, bubble plot analyses indicated that CD8_Tex_GZMB^+^LAG3^+^ cells engaged in antigen presentation–related signaling pathways (including HLA-A–CD8B, HLA-B–CD8B, and HLA-C–CD8B) with other T cell subsets, consistent with ongoing antigen exposure and potential antigen-driven activation (**Figure 4C**). Collectively, these results delineate the transcriptional, functional, and communication characteristics of CD8_Tex_GZMB^+^LAG3^+^ cells, providing insight into their distinct role in the immune landscape of sepsis.

### 3.4 ZNF683 is progressively upregulated along the inferred exhaustion trajectory of CD8^+^GZMB^+^ T cells in sepsis.

**Figure 5:**
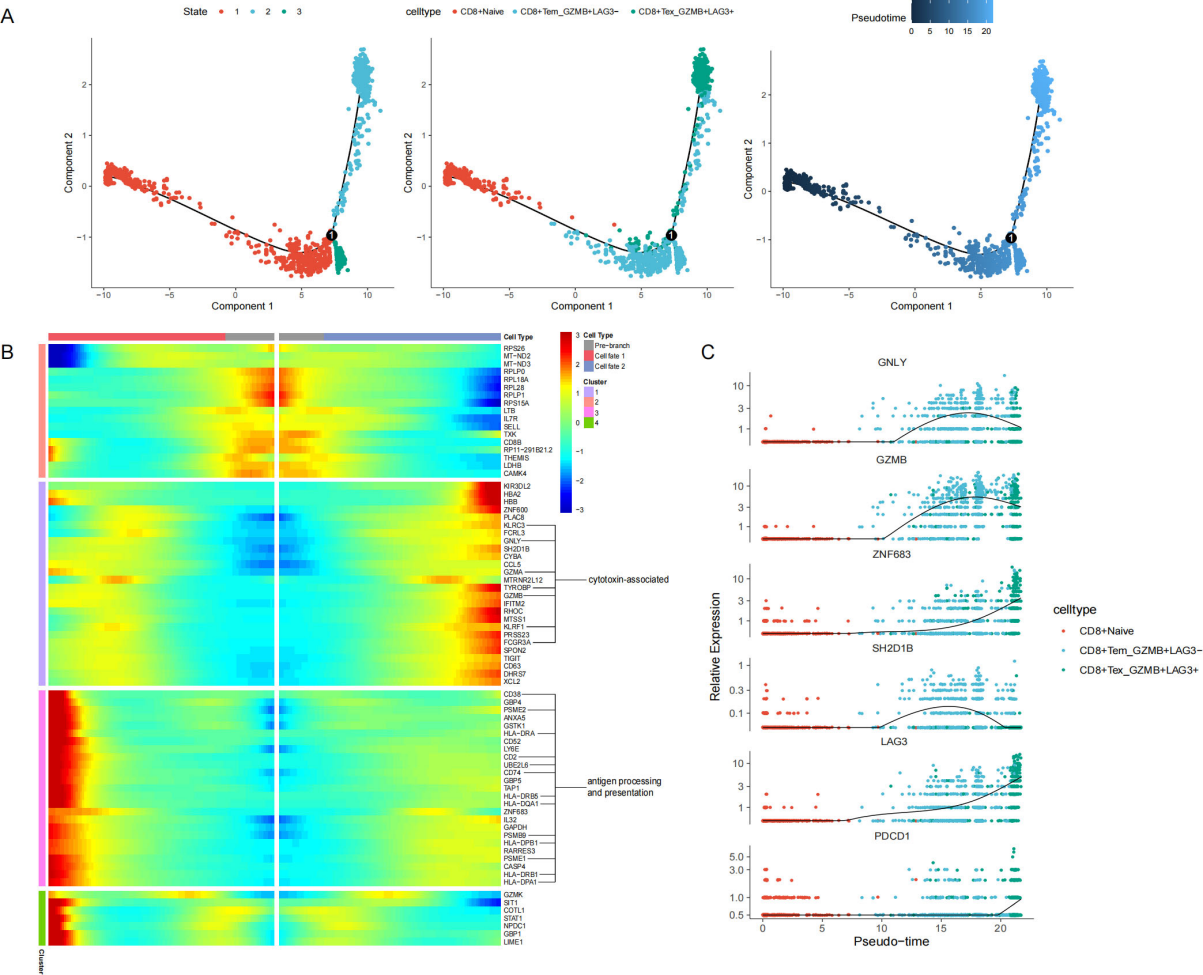
**(A)** Trajectory analysis of CD8_Naive, CD8_Tem_GZMB^+^LAG3^−^, and CD8_Tex_GZMB^+^LAG3^+^ cells performed using Monocle 2. The reconstructed trajectories reveal three differentiation stages—Naive, Tem, and Tex—representing the progressive transition of CD8^+^ T cells. **(B)** Heatmap displaying the scaled expression of differentially expressed genes (DEGs) across the three stages shown in **(A)**. Several gene clusters are enriched in pathways related to antigen processing and presentation and cytotoxic effector function, as determined by Gene Ontology (GO) analysis. **(C)** Gene expression dynamics of representative markers along pseudotime, illustrating the transition from CD8_Naive cells bifurcating into CD8_Tem_GZMB^+^LAG3^−^ and CD8_Tex_GZMB^+^LAG3^+^ lineages.

To investigate the exhaustion trajectory of CD8^+^GZMB^+^ T cells during sepsis, pseudotime analysis was performed on three clusters—CD8_Naive, CD8_Tem_GZMB^+^LAG3^−^, and CD8_Tex_GZMB^+^LAG3^+^—using *Monocle 2* (**Figure 5A**). These clusters represented distinct immune states along a continuous differentiation trajectory. CD8_Naive cells were primarily positioned at the early developmental stage, whereas CD8_Tex_GZMB^+^LAG3^+^ cells localized to the terminal exhausted stage.

Branch point analysis at branch 1 along the pseudotime trajectory revealed that cytotoxicity-associated genes (GNLY, GZMA and GZMB) were progressively downregulated toward the CD8_Tex_GZMB^+^LAG3^+^ branch, while antigen processing–related genes (e.g., TAP1, B2M and PSMB9) were upregulated in the same direction (**Figure 5B**). The expression dynamics of representative genes showed that cytotoxicity-related genes were highly expressed in CD8_Tem_GZMB^+^LAG3^−^ cells and markedly decreased in CD8_Tex_GZMB^+^LAG3^+^ cells, whereas exhaustion markers (LAG3, PDCD1) were increased in the terminal exhausted subset (**Figures 5B, C**).

Along the pseudotime trajectory, the transcription factor ZNF683 displayed a continuous upregulation trend toward the CD8_Tex_GZMB^+^LAG3^+^ state, while the adaptor protein SH2D1B (EAT-2) was downregulated. Visualization of gene expression patterns confirmed that ZNF683 expression increased concomitantly with the exhaustion process, whereas SH2D1B expression peaked in CD8_Tem_GZMB^+^LAG3^−^ cells and declined toward the exhausted population.

Collectively, the pseudotime analysis delineates a transcriptional continuum from naïve to effector to exhausted CD8^+^ T cells in sepsis, accompanied by decreased cytotoxicity, enhanced antigen presentation, and dynamic modulation of regulatory genes such as ZNF683.

### 3.5 LAG3 blockade modulates exhausted-like CD8^+^ T-cell features and is accompanied by reduced tissue ZNF683 signal in the CLP model

**Figure 6:**
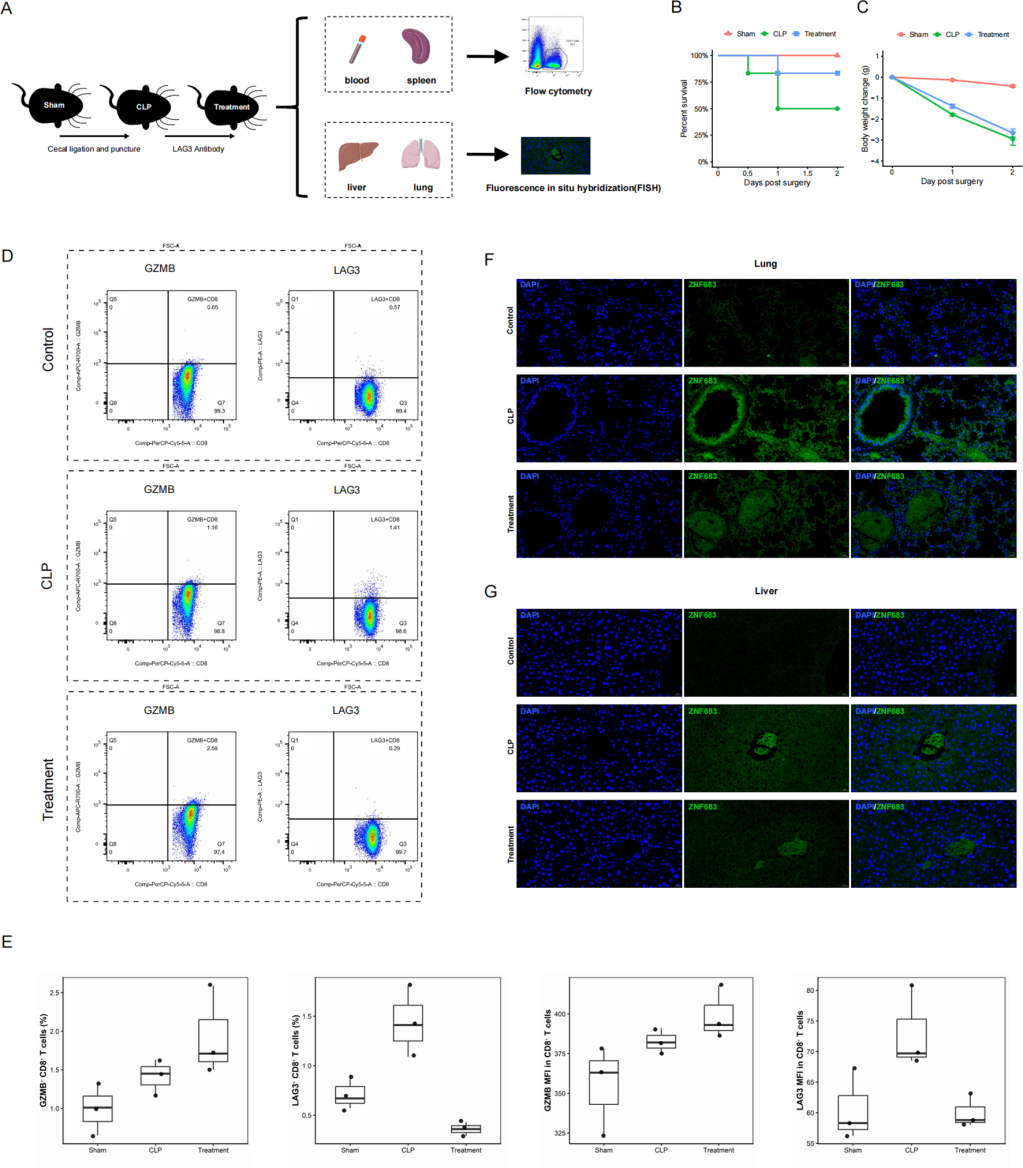
**(A)** Experimental design of the cecal ligation and puncture (CLP) model. Mice underwent CLP, sham laparotomy, or peri-CLP anti-LAG3 treatment (3 consecutive days of anti-LAG3 administration prior to CLP and continued for 2 days after CLP), and all received fluid resuscitation to establish a clinically relevant model of sepsis. Immune responses were analyzed 48h after surgery. **(B)** Kaplan–Meier survival curves for mice subjected to sham surgery (Sham), cecal ligation and puncture (CLP), or CLP plus anti-LAG3 treatment (Treatment) over the 48-h observation window. Group differences were assessed using the log-rank test (three-group comparison: *p* = 0.1030). **(C)** Body weight change relative to baseline (Day 0) on postoperative Days 1 and 2 in the Sham, CLP, and Treatment groups. Data are presented as mean ± SEM. Group effects were analyzed using a linear mixed-effects model (mouse ID as a random effect; Kenward–Roger degrees of freedom), followed by Benjamini–Hochberg–adjusted *post hoc* comparisons at each time point. Day 1: Sham vs CLP, *p* < 0.0001; Sham vs Treatment, *p* < 0.0001; CLP vs Treatment, *p* = 0.0100. Day 2: Sham vs CLP, *p* < 0.0001; Sham vs Treatment, *p* < 0.0001; CLP vs Treatment, *p* = 0.0635. Body weight was not measured after death. **(D)** Representative flow cytometry plots showing GZMB and LAG3 expression in CD8^+^ T cells from spleen and peripheral blood, gated on CD45^+^CD3^+^CD8^+^ cells. **(E)** Box plots summarizing GZMB and LAG3 expression levels in CD8^+^ T cells among the Sham, CLP, and Treatment groups. **(F, G)** Representative immunofluorescence images of liver and lung tissues co-stained with DAPI (blue) and ZNF683 (green), with merged images shown for the Sham, CLP, and Treatment groups. ZNF683 fluorescence is presented as a tissue-level signal and does not on its own establish cell-type specificity. Magnification: ×40; scale bar: 20 µm.

Previous studies have reported that CD8^+^ T cell exhaustion, characterized by reduced cell numbers and impaired effector function, is associated with adverse outcomes in sepsis. To investigate this phenomenon experimentally, we examined the GZMB^+^LAG3^+^CD8^+^ T cell population across varying degrees of exhaustion using a cecal ligation and puncture (CLP) model of sepsis (38, 39). Male C57BL/6 mice were subjected to CLP, sham laparotomy, or peri-CLP anti-LAG3 treatment, with all groups receiving standard postoperative supportive care (e.g., warming and fluid resuscitation as required) to maintain animal welfare and model stability (**Figure 6A**).

To further contextualize the CLP model and treatment effects, we summarized mouse body weight and survival outcomes and visualized these endpoints (**Figures 6B, C**; **Supplementary Table 3**). Over the 48-h observation period, Kaplan–Meier analysis indicated a trend toward improved survival in the anti-LAG3–treated group compared with CLP alone; however, the overall three-group log-rank test did not reach statistical significance (p = 0.1030; **Figure 6B**).

For postoperative body weight change (**Figure 6C**), we applied a linear mixed-effects model with Benjamini–Hochberg adjustment for *post hoc* comparisons. Body weight change differed significantly between Sham and both CLP and Treatment groups (Sham vs CLP, p < 0.0001; Sham vs Treatment, p < 0.0001). Moreover, the CLP group showed a significant difference versus the Treatment group on postoperative day 1 (p = 0.0100), while the between-group difference on day 2 showed a borderline trend (p = 0.0635).

As LAG3 is known to drive and maintain T cell exhaustion in tumors and chronic infections (23, 40), we evaluated whether its blockade could modulate CD8^+^ T cell dysfunction in sepsis. Flow cytometry analysis of viable CD45^+^CD3^+^CD8^+^ leukocytes isolated from peripheral blood and spleen revealed that the frequency of LAG3^+^CD8^+^ T cells was significantly higher in CLP mice compared with both the sham-operated controls and the anti-LAG3–treated group (Kruskal–Wallis test, p = 0.027) (**Figures 6D, E**). For GZMB^+^CD8^+^ T cells, although the proportion was higher in anti-LAG3–treated mice than in untreated CLP mice, the overall difference did not reach the conventional threshold for statistical significance (Kruskal–Wallis test, p = 0.066). Regarding the MFI of both GZMB and LAG3, similar group-wise patterns were observed (**Figure 6E**).

To assess tissue-level ZNF683 RNA signals in sepsis, fluorescence *in situ* hybridization (FISH) was performed on liver and lung tissues from the Sham, CLP, and Treatment groups. These signals are tissue-level and are not cell-type-specific. As shown in **Figures 6F, G** and **Supplementary Figures 2**-**3**, minimal ZNF683 expression was detected in sham-operated mice, whereas it was upregulated in CLP-induced septic mice. Notably, anti-LAG3 treatment was associated with a reduction in ZNF683 signal intensity in both the liver and lung compared with CLP mice. Collectively, these results show that CLP-induced sepsis was associated with increased LAG3 expression on CD8^+^ T cells and increased tissue ZNF683 signal in liver and lung, whereas anti-LAG3 treatment partially attenuated these changes, consistent with partial reversal of an exhausted-like phenotype.

## 4 Discussion

The 28-day survival rate has traditionally served as a key endpoint for evaluating therapeutic efficacy in sepsis. With improvements in early recognition and intensive early-phase management, more patients now survive the acute phase. Accordingly, increasing attention has shifted toward long-term outcomes, particularly among patients who develop chronic critical illness (CCI), in whom persistent immune dysregulation is thought to contribute to late-phase morbidity and mortality (41).

Sepsis is associated with extensive immune cell apoptosis and depletion of lymphocyte pools, compromising host defense. In the single-cell RNA sequencing dataset (GSE175453), we observed a marked reduction in multiple T-cell subsets, consistent with a profound immunodeficient state in late-stage sepsis. Beyond lymphopenia, functional impairment of the remaining T cells is likely to contribute to late-phase immune dysfunction, and T-cell exhaustion represents a plausible mechanism. We acknowledge that the human dataset represents late-stage sepsis (Day 14–21 post-sepsis), whereas our CLP experiments capture an acute-phase time window (48 h after surgery). Therefore, cross-species comparisons should be interpreted as convergent signals of exhaustion-like programs rather than direct temporal equivalence.

Using reference mapping and single-cell clustering, we classified T cells into transcriptionally distinct subsets and found that CD8+ GZMB+ cells were preferentially represented in sepsis samples. This may seem inconsistent with sepsis-related T-cell loss. However, this is likely attributable to technical sampling rather than true expansion. The 10x Genomics platform processes a limited number of captured cells, and UMAP visualizations represent the composition of the processed cell pool rather than absolute *in vivo* cell numbers. Therefore, this pattern should be interpreted as a relative shift within the profiled T-cell population, not as evidence of increased absolute CD8^+^GZMB^+^ counts. Building on this, we performed a focused analysis of CD8^+^GZMB^+^ cells and resolved two subpopulations with distinct transcriptional profiles. Differential analysis highlighted LAG3 emerged as a key distinguishing feature. Given the established role of LAG3 in T-cell dysfunction in chronic infection and cancer, the preferential representation of a CD8_Tex_GZMB^+^LAG3^+^ subset in late-stage sepsis supports a potential link to immune suppression in this cohort.

Functional annotation of CD8_Tex_GZMB^+^LAG3^+^ cells supported an exhaustion-like remodeling pattern. GO/KEGG analyses indicated enrichment of signatures related to antigen presentation and MHC class I–associated binding, alongside oxidative phosphorylation and ROS-linked pathways. In parallel, genes involved in cytotoxic effector activity and immune activation were relatively reduced. Together, these features are consistent with sustained antigen engagement accompanied by attenuated cytolytic function and altered bioenergetic demands. This interpretation is biologically plausible in sepsis, where mitochondrial dysfunction and excessive ROS generation are prominent and may contribute to impaired immune competence (42, 43). Although mitochondrial-targeted interventions have been reported to modulate T-cell function in other settings (44), the contribution of metabolic stress and oxidative pathways to lymphocyte exhaustion during late-stage sepsis remains insufficiently defined (45).

We next considered how these observations relate to cytotoxic and exhausted states described in other disease settings. Tumor studies have reported highly cytotoxic Ib-CD8^+^ T cells activated through MHC class I pathways in a T-bet–dependent manner (46). The transcriptional profile of these Ib-CD8^+^ T cells share similarity with our identified CD8^+^GZMB^+^ population. However, in late-stage sepsis, the LAG3-high subset exhibited concomitant upregulation of antigen presentation–related genes and downregulation of cytotoxic and immune activation genes, suggesting persistent antigen stimulation coupled with impaired effector output. Sustained antigen engagement in sepsis may be facilitated by enhanced cross-presentation; for example, platelet-associated MHC-I upregulation has been proposed to promote prolonged antigen-specific CD8^+^ stimulation and subsequent functional impairment (47). Although we did not directly test these upstream mechanisms, they provide a plausible framework for the persistence of LAG3-high dysfunctional CD8^+^ states.

Consistent with this framework, cytotoxic gene set scoring indicated that CD8_Tex_GZMB^+^LAG3^+^ cells retained residual cytotoxic potential but had lower cytotoxicity scores than CD8_Tem_GZMB^+^LAG3^−^ cells. This supports the view that exhaustion reflects a graded decline in effector competence rather than an all-or-none loss of cytotoxic machinery. PDCD1 (PD-1) was detectable but did not significantly distinguish these subsets (48). Given that PD-1 is widely used as a marker of exhaustion across chronic infections, cancer, and autoimmune disease (49), this finding suggests that sepsis-associated CD8^+^ T-cell dysfunction may be better viewed as a continuum of transcriptional and metabolic states driven by persistent antigen exposure and systemic stress. In this setting, LAG3 may serve as a more informative marker of an exhausted-like state than PD-1 in late-stage sepsis.

Cell–cell communication analysis suggested that CD8_Tex_GZMB^+^LAG3^+^ cells engaged in fewer and weaker predicted ligand–receptor interactions than other T-cell subsets, consistent with a less interactive immune profile, as restoring productive interactions between exhausted T cells and dendritic cell subsets has been associated with T-cell reinvigoration in oncology (50). CellChat also indicated stronger antigen presentation–related signals in CD8_Tex_GZMB^+^LAG3^+^ cells than in CD8_Tem_GZMB^+^LAG3^−^ cells, supporting a role for sustained antigenic stimulation in maintaining exhaustion-like programs in sepsis.

Pseudotime ordering was consistent with a gradual transition from CD8_Tem_GZMB^+^ to CD8_Tex_GZMB^+^LAG3^+^ cells during sepsis. Branch point analysis further identified distinct transcriptional programs along the two trajectories, involving genes beyond those linked to cytolysis and canonical exhaustion programs. Among candidate regulators, ZNF683 showed a progressive increase along the inferred exhaustion continuum, indicating that its expression tracks with this transition. Prior single-cell studies reported expansion of ZNF683^+^ CD8^+^ memory-like subsets in sepsis and enrichment of oxidative phosphorylation and ROS-associated pathways (51), supporting a potential connection between ZNF683 expression and metabolic remodeling. In other disease models, ZNF683 has been linked to exhaustion-like profiles in cytotoxic lymphocytes; for example, ZNF683 overexpression in NK cells was reported to suppress SH2D1B transcription via promoter binding, leading to impaired cytotoxicity and acquisition of an exhaustion-like state (52). Together, these observations support the hypothesis that ZNF683 may participate in regulatory circuits connecting metabolic reprogramming and reduced cytotoxic signaling in CD8^+^GZMB^+^ T cells during sepsis. Nevertheless, ZNF683 upregulation could also reflect a compensatory response to persistent antigen stimulation and mitochondrial stress rather than serving as a primary driver.

Our *in vivo* experiments in CLP-induced septic mice provided supportive evidence for exhaustion-associated features in GZMB^+^CD8^+^ T cells and suggested that LAG3 blockade may partially improve cytotoxic competence. Anti-LAG3 treatment was associated with an increased frequency of GZMB^+^CD8^+^ T cells compared with untreated CLP mice, consistent in direction with prior reports that LAG3 deficiency or blockade can improve survival and bacterial clearance in CLP models. While statistical support was limited—likely due to sample size—the directionality of change is biologically plausible and supports further evaluation of LAG3 as a checkpoint axis in sepsis-associated CD8^+^ T-cell dysfunction. In parallel, ZNF683 was highly expressed within the exhausted GZMB^+^LAG3^+^CD8^+^ T-cell state in the single-cell analysis, and RNA FISH demonstrated elevated ZNF683 signal in organs from CLP mice, with reduced expression following anti-LAG3 treatment. These observations suggest that ZNF683 expression may track with LAG3-associated dysfunctional states *in vivo*.

ZNF683 has been described as a marker of tumor-reactive CD8^+^ T-cell populations across several cancer microenvironments and has also been linked in promoting CD8^+^ T-cell proliferation and IFN-γ secretion in the AIDS model of SIVmac239 infection, underscoring context-dependent functions. One possibility is that ZNF683 supports cytotoxic differentiation during acute activation, whereas sustained in the setting of prolonged antigen exposure and metabolic stress—as may occur in late-stage sepsis—could coincide with dysfunctional remodeling. This potential nonlinearity highlights the need to define the temporal dynamics of ZNF683 and its downstream regulatory network to determine whether it functions as a driver, a permissive factor, or a marker of exhaustion progression in sepsis.

In summary, our study identifies an exhausted-like CD8_Tex_GZMB^+^LAG3^+^ T-cell subset preferentially represented in late-stage sepsis and integrates transcriptomic, computational, and *in vivo* approaches to explore mechanisms of persistent immune dysfunction. The data are consistent with a model in which sustained antigenic stimulation and metabolic remodeling are associated with progressive attenuation of CD8^+^ cytotoxic function, with ZNF683 emerging as a candidate transcriptional regulator linked to this trajectory. These findings motivate further mechanistic and translational research into checkpoint pathways and transcriptional regulators as potential entry points to mitigate sepsis-associated immunosuppression.

Several limitations warrant consideration. First, the human single-cell analysis relied on publicly available datasets with limited sample size and incomplete clinical metadata, restricting adjustment for confounding and potentially limiting generalizability. Validation in larger, prospectively collected, well-phenotyped cohorts will be important, and donor-aware strategies will be essential in future studies with larger cohorts. Second, although the CLP model recapitulates key aspects of sepsis, it cannot fully reflect the heterogeneity and prolonged immunopathology observed clinically; multi-timepoint *in vivo* sampling and complementary models may help define stage-specific exhaustion programs. Third, our study did not directly manipulate ZNF683, and therefore causality and downstream regulatory mechanisms remain unresolved. In addition, our tissue RNA FISH provides organ-level signals without CD3/CD8 co-localization; cell-type specificity will require CD3/CD8 co-staining or assays on sorted CD8^+^ T cells with quantitative readouts. Follow-up studies should employ loss- and gain-of-function approaches (e.g., conditional knockout) combined with chromatin-level profiling to define ZNF683 targets and to test whether modulating ZNF683 alters exhaustion phenotypes and infection susceptibility in late-stage sepsis.

## 5 Conclusion

We identified an exhaustion-like LAG3^+^GZMB^+^ CD8^+^ T-cell subset enriched in late-stage sepsis, characterized by reduced cytotoxic signatures and increased inhibitory-receptor expression. ZNF683 was consistently elevated in this state and increased along the inferred CD8^+^GZMB^+^ exhaustion trajectory. In the CLP model, LAG3 blockade partially shifted CD8^+^ T-cell features toward improved effector profiles and was accompanied by reduced tissue ZNF683 signals, suggesting that ZNF683 tracks with sepsis-associated CD8^+^ T-cell dysfunction. Together, these findings highlight ZNF683 as a marker linked to exhaustion-like CD8^+^ T-cell states in sepsis and warrant direct perturbation studies to test its causal role and therapeutic potential.

## Data Availability

The scRNA-seq dataset analysed in this study is publicly available in GEO under accession GSE175453. The original contributions presented in the study are included in the article/**Supplementary Material**. Further inquiries can be directed to the corresponding authors.
