# Supplementary material for: The transcription factor ZNF683 marks an exhaustion-like GZMB+CD8+ T cell in sepsis
Source: Front Immunol. 2026 Mar 6;17:1756339. doi: 10.3389/fimmu.2026.1756339 (PMC13002821; doi:10.3389/fimmu.2026.1756339)
Supplement: Supplementary file 1 [file DataSheet1.docx]

Supplementary Material

## Supplementary Figures

##
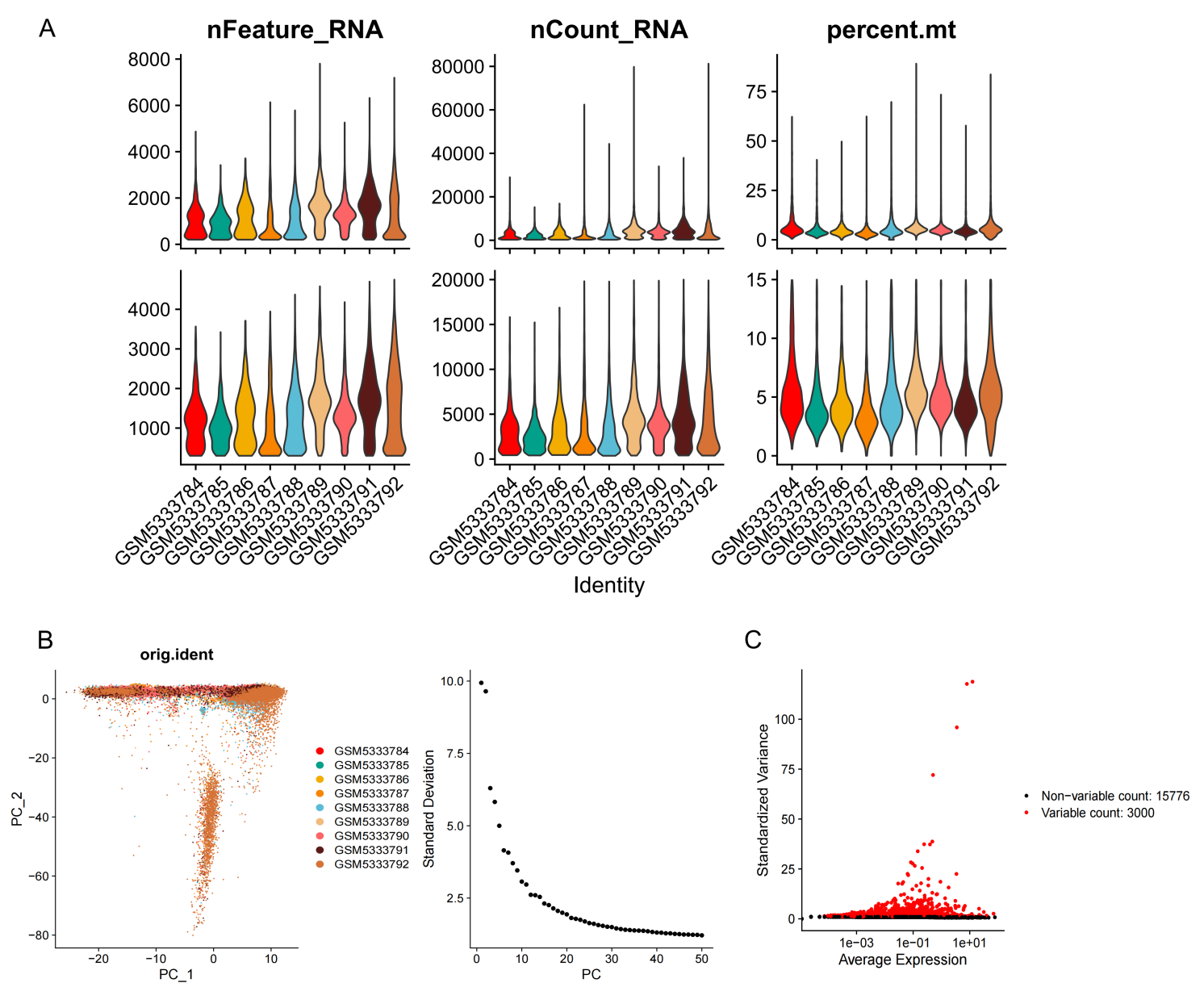


**Supplementary Figure 1.** (A) Violin plots showing the distribution of the number of detected genes (nFeature_RNA), total UMI counts (nCount_RNA), and mitochondrial gene percentage (percent.mt) across the nine samples (GSM5333784–GSM5333792). (B) Principal component analysis (PCA) of all cells colored by sample identity (left) and elbow plot of the standard deviation for each principal component used to determine the number of PCs included in downstream analysis (right). (C) Mean–variance plot displaying standardized variance as a function of average gene expression, with highly variable genes (Variable count = 3000) highlighted against non-variable genes (Non-variable count = 15776).

##
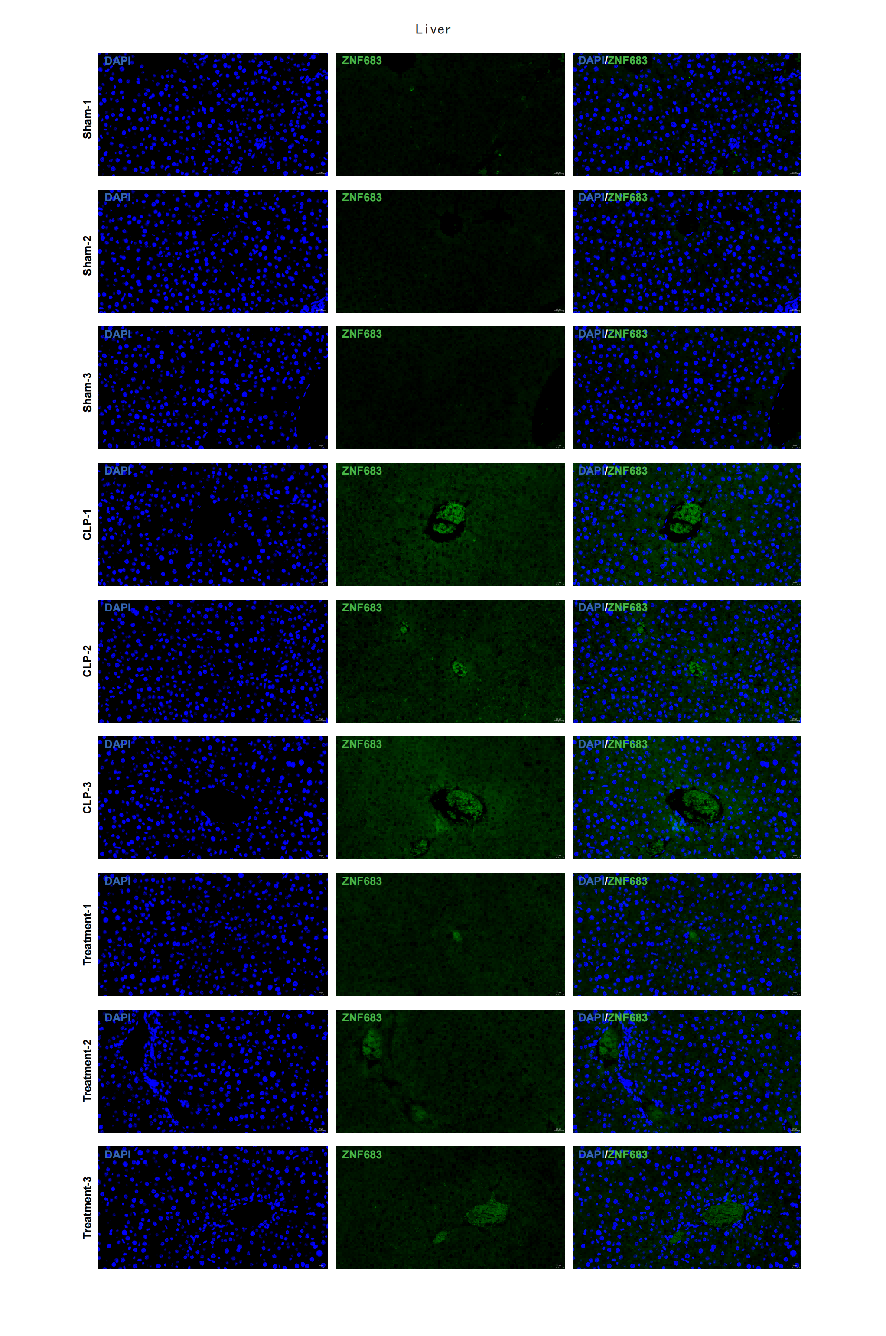


**Supplementary Figure 2.** Representative RNA-FISH images of ZNF683 expression in the liver .


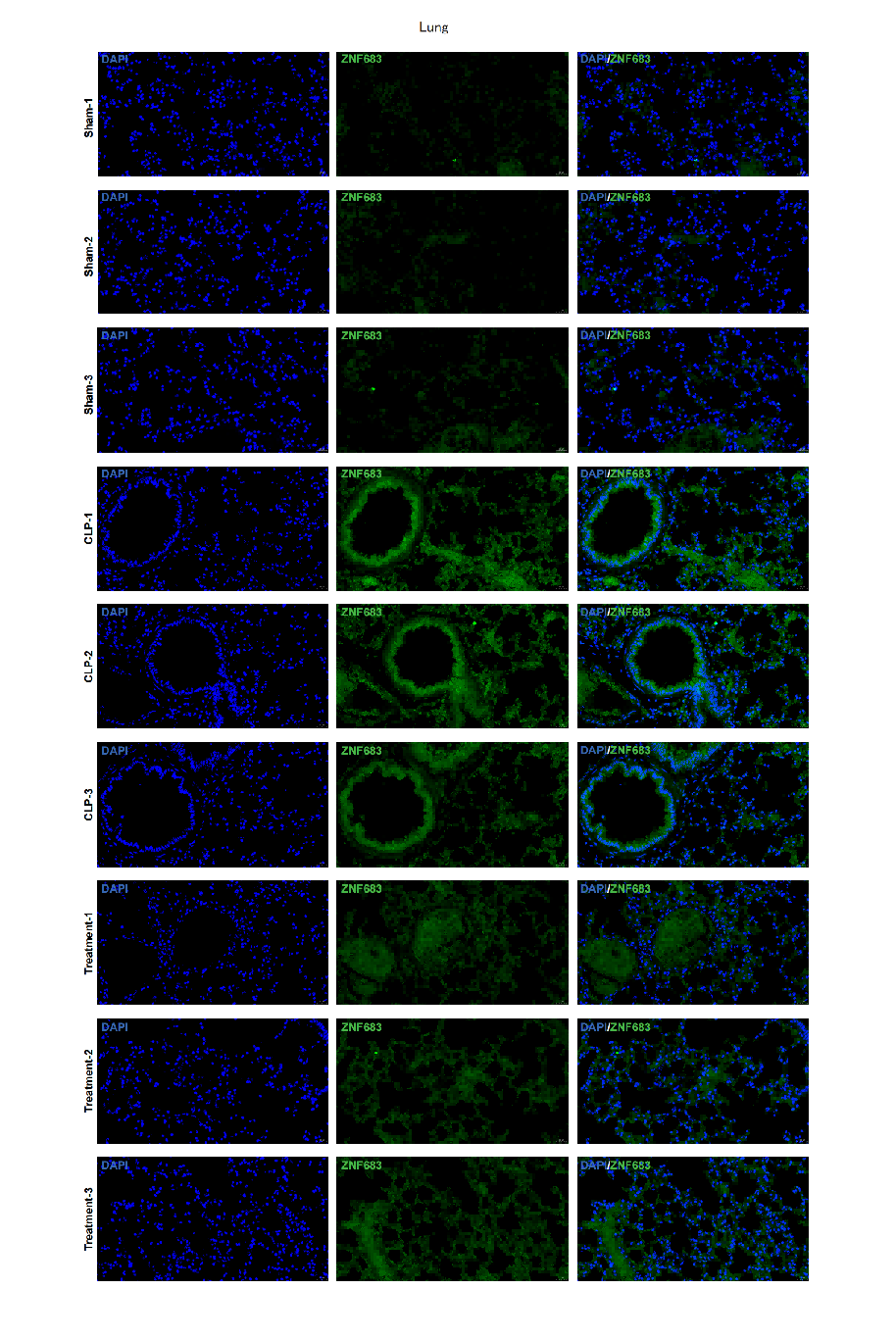


**Supplementary Figure 3.** Representative RNA-FISH images of ZNF683 expression in the lung .

## Supplementary Table

|  | Sample name | GZMB+ (%CD8) | LAG3+ (%Cd8) | GZMB (MFI) | LAG3 (MFI) |
| --- | --- | --- | --- | --- | --- |
| Treatment | 773_017.fcs | 2.59% | 0.29% | 418 | 58 |
|  | 774_002_004.fcs | 1.71% | 0.36% | 393 | 58.8 |
|  | 775_005.fcs | 1.50% | 0.43% | 386 | 63.1 |
| Sham | 781_023.fcs | 0.65% | 0.57% | 323 | 56.2 |
|  | 782_011.fcs | 1.01% | 0.91% | 363 | 67.3 |
|  | 783_026.fcs | 1.31% | 0.67% | 378 | 58.3 |
| CLP | 788_008.fcs | 1.45% | 1.81% | 383 | 80.9 |
|  | 791_020.fcs | 1.16% | 1.41% | 375 | 69.7 |
|  | 776_014.fcs | 1.63% | 1.09% | 391 | 68.5 |

**Supplementary Table S1.**Splenic CD8⁺ T cells from sham-operated mice (Sham), CLP-induced septic mice (CLP), and CLP mice treated with anti-LAG3 antibody (Treatment) were analyzed by flow cytometry.

| GEO | Group | Sepsis type | Clinical phenotype | Primary sepsis diagnosis | SOFA Score | APACHE II Score |
| --- | --- | --- | --- | --- | --- | --- |
| GSM5333784 | Healthy control | Healthy Control | Healthy Control |  |  |  |
| GSM5333785 | Healthy control | Healthy Control | Healthy Control |  |  |  |
| GSM5333790 | Healthy control | Healthy Control | Healthy Control |  |  |  |
| GSM5333791 | Healthy control | Healthy Control | Healthy Control |  |  |  |
| GSM5333792 | Healthy control | Healthy Control | Healthy Control |  |  |  |
| GSM5333786 | Late-stage sepsis | Bacterial | Rapid Recovery | Necrotizing Soft Tissue Infection | 7 | 13 |
| GSM5333787 | Late-stage sepsis | Fungal | Chronic critical illness | Fungemia | 10 | 21 |
| GSM5333788 | Late-stage sepsis | Bacterial | Chronic critical illness | Surgical Site Infection- Implant/graft infection |  |  |
| GSM5333789 | Late-stage sepsis | Fungal | Chronic critical illness | Surgical Site Infection- Implant/graft infection |  |  |

**Supplementary Table S2.** Clinical and sample metadata for the GSE175453 human cohort.
This table summarizes GEO sample accessions, group assignment, sepsis type, clinical phenotype, and the primary sepsis diagnosis for septic donors. Where available, severity indices at enrollment are provided, including the SOFA score and APACHE II score. Blank cells indicate that the corresponding information was not reported in the publicly available metadata for that sample.

| Mouse ID | group | outcome | weight.0 | weight.1 | weight.2 | death time |
| --- | --- | --- | --- | --- | --- | --- |
| 781 | Sham | Survived | 23.85 | 23.69 | 23.34 |  |
| 782 | Sham | Survived | 25.68 | 25.53 | 25.02 |  |
| 783 | Sham | Survived | 24.5 | 24.39 | 24.19 |  |
| 784 | Sham | Survived | 24.78 | 24.64 | 24.42 |  |
| 785 | Sham | Survived | 24.87 | 24.76 | 24.52 |  |
| 786 | Sham | Survived | 24.3 | 24.18 | 23.95 |  |
| 776 | CLP | Survived | 25.76 | 23.97 | 22.46 |  |
| 787 | CLP | Died | 25.17 |  |  | 24 |
| 788 | CLP | Survived | 24.87 | 23 | 21.68 |  |
| 791 | CLP | Survived | 23.6 | 21.89 | 21.26 |  |
| 792 | CLP | Died | 25.27 |  |  | 12 |
| 793 | CLP | Died | 24.74 |  |  | 24 |
| 771 | Treatment | Survived | 24.77 | 23.31 | 21.86 |  |
| 772 | Treatment | Died | 26.39 |  |  | 24 |
| 773 | Treatment | Survived | 25.47 | 24.13 | 22.63 |  |
| 774 | Treatment | Survived | 26.44 | 24.79 | 23.56 |  |
| 775 | Treatment | Survived | 23.57 | 22.15 | 20.83 |  |
| 777 | Treatment | Survived | 24.57 | 23.53 | 22.66 |  |

**Supplementary Table S3.** This table summarizes individual mouse identifiers, group assignment, survival outcome, body weight measurements, and time-to-death information. weight.0, weight.1, and weight.2 denote mouse body weight (g) measured at the time of sham surgery or CLP induction (day 0) and on postoperative days 1 and 2, respectively. Death time refers to the elapsed time from sham surgery or CLP to death, recorded in 12-hour intervals. Body weight was not measured after death.
